# Evaluating the Efficacy of Employing Local Anesthetic Prostatic Blocks During Rezum Procedure

**DOI:** 10.7759/cureus.29598

**Published:** 2022-09-26

**Authors:** Leigh Gilpin, David Zekan, Bryce Baugh, Apexa Patel, Stacie A Deslich, Samuel Deem, Ryan Fitzwater, Joshua Lohri, James Tierney, Nathan E Hale

**Affiliations:** 1 Department of Urology, Charleston Area Medical Center, Charleston, USA; 2 Research Urology, Charleston Area Medical Center, Center for Health Services and Outcomes Research, Charleston, USA; 3 Research, Charleston Area Medical Center, Center for Health Services and Outcomes Research, Charleston, USA

**Keywords:** benign prostatic hyperplasia, pain management, prostate block, prostatic hyperplasia, rezum therapy

## Abstract

Introduction

Rezum is a minimally invasive, outpatient procedure using convective water vapor to relieve outlet obstruction from benign prostatic hyperplasia (BPH). Evidence on the technical approach of Rezum therapy, particularly pain control, is lacking. The purpose of this study was to evaluate the efficacy of utilizing a local anesthetic prostate block for postoperative pain control during Rezum therapy for BPH. A multimodal approach is typically utilized for pain control during and after Rezum. However, little is known about which elements are most critical.

Methods

This is a single-center retrospective study of 109 patients who underwent Rezum for BPH. Patients were then divided into two groups: Local anesthetic prostatic block verse no local anesthetic prostatic block for the procedure. A phone survey was performed to assess the patients' subjective pain scores and postoperative analgesics usage. A comparison of reported pain scores on a 0-10 Likert scale as well as usage of prescription and non-prescription analgesics medications was performed.

Results

There were 109 patients who underwent Rezum therapy, and 86 (79%) of patients responded to phone surveys. There was no significant difference in postoperative pain scores between patients who received local anesthetic prostatic block vs those who did not (2.10 vs 3.03). Similarly, there were no significant differences in postoperative narcotics or non-prescription analgesic medications usage.

Conclusion

Our data suggest that when performing Rezum using conscious sedation in the operating room or cystoscopy suite, it is unnecessary to perform a local anesthetic prostate block as it has no significant effect on patient-reported pain or the use of analgesics in the postoperative period.

## Introduction

Approximately 70% of men have some evidence of benign prostatic hyperplasia (BPH), by the age of 70 [1]. The degree of hyperplasia correlates strongly with the development of lower urinary tract symptoms (LUTS), which affect both quality of life and voiding function in these patients. Various treatment modalities for BPH exist, including medications, transurethral resection of the prostate (TURP), open prostatectomy, and more recently effective minimally invasive modalities. Differences in efficacy and invasiveness make an individualized approach to treatment crucial. One of the newer treatment modalities is Rezum convective water therapy.

Rezum using convective water therapy is a minimally invasive, outpatient procedure designed to relieve obstructive symptoms associated with BPH [2]. Rezum is commonly performed in an office or ambulatory outpatient surgery center. An important aspect of this procedure is the management of patients’ discomfort and anxiety. Previous studies have described using a combination of therapies including oral sedation, intravenous sedation, periprostatic nerve blocks, and post-treatment analgesics [1-4]. In pilot studies using Rezum, sedation was predominantly given orally (69%), but some intravenous sedation was utilized (21%) [1,4]. Anti-inflammatories and urinary analgesics/anti-spasmodics have been documented in multimodal pain management plans [3]. Local anesthesia prostatic blocks have been performed as well [1,4]. 

Evidence on the best technical approach to managing pain related to Rezum therapy is lacking. Currently, large variations in pain management exist amongst practitioners when performing Rezum. Published recommendations are based on urologist experience, without a comparison of the efficacy of opposing methods [1-5]. We sought to assess the efficacy of using local anesthesia when performing Rezum under intravenous sedation. One of our treatment strategies was to offer a minimally invasive procedure that offered minimal pain, anxiety, and a smooth postoperative course. We analyzed patient-reported post-operative pain scores and the use of analgesics between groups who did and did not receive a local anesthesia prostatic block.

## Materials and methods

Data and study population

Following the approval of the institutional review board, a single-institution retrospective chart review accompanied by a phone survey of 109 men was performed who underwent Rezum therapy between January 1, 2017, and April 20, 2019. Data pertaining to age, BMI, prostate size, international prostate symptoms scores, post-void residuals on bladder scans, and a number of treatment sites were gathered by retrospective chart review. Foley size, length of time, and median lobe were not recorded from the scan. Pain scores and medication usage was obtained by the phone survey.

A total of 86 patients out of 109 willingly provided verbal informed consent and participated in the phone survey. During the survey, the surveyor read the patient the pain scale and the patient reported their pain level. Data on prescriptions filled and usage of pain medication post-operatively were collected through retrospective chart review and survey questions. The patients were divided into two groups: one was with local anesthetic prostatic block prior to Rezum therapy and the control group was without local anesthetic prostatic block prior to therapy. This study was non-blinded and included no use of placebos. Survey questions can be found in the first table of the Appendices section. 

Statistical analysis

Comparison of reported pain scores (on a 0-10 scale) [6] and usage of prescription and non-prescription pain medications were performed between groups. Data analysis was performed using SAS 9.4. Descriptive analysis included means, standard deviations, ranges, and percentages being reported for the respective variables. Continuous variables were analyzed using the Student’s t-test and categorical variables using the chi-square and Mann-Whitney U test. All comparisons were performed at 95% of the level of significance and a p-value <0.05. 

Prostate block procedure

The local anesthetic prostatic block was performed via a transperineal route employing a transperineal access system (Precision Point, Perineologic, Cumberland, MD) [7]. The transperineal approach, chosen for its increased safety, delivers a field block that blocks the afferent fibers to the prostate from the pudendal nerve, peroneal nerve, and pelvic plexus [8]. In addition, local anesthesia was injected to include the levator ani muscle, the deep perineal muscles as well as the prostatic capsule. The technique involved to deliver this injection includes a linear array ultrasound probe placed in the rectum, with a transperineal access system applied to the ultrasound probe [9]. Two transperineal needle sticks 1 cm lateral to the midline on the left and right sides. We used a 7-inch 22 gauge needle and advanced through the transperineal access system to deliver the local anesthetics i.e., a 50:50 combination of 1% xylocaine plain and 0.5% Marciane plain. A total of 10 ml mixture was used with 5 ml being delivered to each side. Two ml was injected into the levator muscle and the remaining local was deposited in the space between the pelvic diaphragm and the prostatic apex creating the desired field block. The pudendal block was not given. With the ultrasound probe still in the rectum, the prostate measurements were then taken. Once the local anesthesia and prostate measurement was completed, a cystoscopy was performed to evaluate and measure the prostatic urethra. The Rezum procedure was then performed. Post-procedure, a 16 Fr Foley catheter was placed and remained for three days.

## Results

Of the 109 patients who underwent Rezum therapy, 86 (79%) of patients responded to phone surveys. The following means were reported: mean age of patients participating in this study was 68.5 years (48-95), mean BMI was 29.4 kg/m2 (18-39.7), mean prostate size was 51.5 g (18-170), mean pre-treatment was IPSS 10 (0-31), mean pre-treatment was PVR 141 mL (0-990 mL), and the mean number of treatments was five (2-10). No significant differences in age (69.2 vs. 67.8 years) or BMI (28.95 vs. 29.84 kg/m2) exist between groups who did and did not receive a local anesthetic prostatic block. Similarly, no differences existed in prostate size (49.47 vs. 47.12 g), treatment number (5.02 vs. 4.4), pre-treatment IPSS (10.2 vs. 9.4), or pre-treatment PVR (146.9 vs. 135.9 mL) between groups (Table 1). The International Prostate System Score (IPSS) is found in the second table in the Appendices section.

**Table 1:** Demographic data and pre-treatment values for patients undergoing Rezum therapy with no significant differences between groups with and without the local anesthetic prostatic block BMI: body mass index, IPSS: International Prostate System Score; PVR: post-void residual urine test, SD: standard deviation

	No block (SD)	Block (SD)	P-value
Age (years)	67.79±9.23	69.2±9.35	0.449
BMI (kg/m^2^)	29.84±5.45	28.95±5.23	0.415
Prostate size (g)	47.12±22.53	49.47±21.09	0.658
# treatments	4.4±1.79	5.02±1.87	0.095
Pre-treatment IPSS	10.2±7.5	9.4±7.7	0.51
Pre-treatment PVR (mL)	135.9±159.8	146.9±176.0	0.764

The post-operative pain scores for patients with and without local anesthesia are presented in Table 2, with a mean score of 2.10 in those who received local anesthesia and 3.03 in those who did not. A comparison of pain scores between groups using non-parametric tests showed no statistically significant difference. 

**Table 2:** Patient-reported pain scores and comparisons between groups using Mann-Whitney U-test

	Pain score without block (%) N=50	Pain score with block (%) N=33	P-value
no pain (0)	8 (16)	8(24.24)	0.123
mild (1-3)	21(42)	15(45.45)	
moderate to severe (4-6)	12(24)	9(27.27)	
severe to very severe(7-9)	5(10)	1(3.03)	
worst pain(10)	4(8)	0	

Similarly, prescription and usage of narcotic pain medication and usage of over-the-counter (OTC) pain medication were compared between groups. All patients received the same narcotic and dosage. No significant differences in prescription or usage of medication were shown between patients who did and did not receive local anesthesia prior to Rezum therapy (Table 3).

**Table 3:** Comparison of prescription and use of narcotic and OTC pain medication in patients who did and did not receive the local anesthetic prostatic block OTC: over the counter

	N		No block (%)	Block (%)	Chi 2 p-value
Prescribed narcotics	84	NO	9(18)	6(17.65)	0.436
		YES	28(56)	23(67.65)	
		UNSURE	13(26)	5(14.71)	
Used narcotics	84	NO	28(56)	23(67.65)	0.275
		YES	11(22)	8(23.53)	
		UNSURE	11(22)	3(8.82)	
Used OTC pain medication	82	NO	28(57.14)	18(54.55)	0.588
		YES	12(24.49)	11(33.33)	
		UNSURE	9(18.37)	4(12.12)	

## Discussion

Many different combinations of pain management have been described in the literature [1-5]. Prior to this study, no one had investigated which combination provides the best benefits. We explored the role of using local anesthetic prostatic block in the setting of IV sedation at the time of Rezum therapy for the management of BPH. Our initial hypothesis was that performing a local block during Rezum while using IV sedation would improve reported postoperative pain and narcotic usage. This however was not what our data revealed. Performing a local anesthetic prostatic block during Rezum water therapy did not appear to reduce patient’s reported pain in the postoperative period. Local anesthetic block also failed to reduce the usage of narcotics or OTC pain medications.

The current literature suggests that the decisions to perform Rezum in the office versus an ambulatory surgery setting and management of pain and anxiety should be joint between the patient and his provider [10-12]. A multimodal approach with monitored anesthesia and postoperative analgesia is generally recommended for pain control [3]. Others have stated that this procedure requires local anesthesia with possible sedation [10]. Our practice within our group through this study was to perform Rezum in an ambulatory surgery center under IV sedation.

Our data suggest using local anesthetic block has no correlation with patient-reported pain or the prescription or use of analgesics (prescription or OTC) post-operatively, suggesting the use of local anesthetic block is extraneous when used in adjunct with IV sedation. Therefore, local anesthesia has limited utility when performed under IV sedation with regard to postoperative pain in patients undergoing Rezum. It is reasonable to assume that a local prostatic block in the absence of IV sedation would reduce postoperative pain; however, this wasn’t assessed in this study. Local anesthetic prostate block has been well-established for pain control for prostate biopsies [13]. Prostatic blocks have also been shown to decrease patients’ reported postoperative pain scores and analgesic use when a periprostatic nerve block was used in combination with transurethral resection of the prostate [14]. This finding contradicts our findings. We believe that they found a benefit because a transurethral resection of the prostate is a more invasive procedure than Rezum, causing significantly more pain. A longer-acting agent may also prove to be useful in managing pain.

Another interesting point is that no patients who received a local block reported a 10 out of 10 pain while four patients in the group without a block reported 10 out of 10 pain. The two groups were not significantly different but clearly, four patients didn’t have a positive experience. As urologists are consistently evaluating ways to improve the patient experience, performing a prostatic block comes at minimal risk and may spare a patient from reporting 10 out of 10 pain, so it does not appear unreasonable to perform a prostatic nerve block.

Overall, 60% of our patients who responded to our survey were prescribed pain medication by their surgeon. Interestingly, only 23% of patients reported using narcotics, and 27% reported using OTC analgesics. The data suggests that we overprescribed narcotics following Rezum, and we have changed our prescribing patterns based on this survey. No statistically significant differences were seen in prescription/use of pain medication or patient-reported pain scores between groups who did and did not receive local anesthetic prostatic block prior to the procedure. The survey data suggested there was a lack of utilization of pain medication post-operatively in both groups. This suggests that perioperative pain is not a big problem with or without local anesthetic blocks.

There are certainly limitations to the study. This was a non-randomized retrospective study and the decision to perform prostatic nerve block was based on surgeon preference. The study may also introduce recall bias as many patients were being surveyed regarding pain that generally resolves quickly in the post-operative period. However, despite the possible recall bias, there is value in reporting how patients’ remember their postoperative experience. Additionally, not all sources of pain were addressed by the survey questions. For example, bladder spasm pain was not discussed.

## Conclusions

Our study suggests that when performing Rezum under IV sedation, it is unnecessary to perform a local anesthetic prostatic block as it has no significant effect on patient-reported pain or the prescription or usage of analgesics post-operatively. The Rezum procedure has been shown to be well tolerated post-operatively. Only 23% of patients used the prescribed narcotics and 27% used OTC pain medications, suggesting that prescribing narcotics following Rezum could be avoided. Rezum appears to provide a reasonable minimally invasive therapy for BPH management.

## Appendices

 Table 4 contains the survey questions of this study.

**Table 4:** Appendix A

	Improved	Unchanged	Worsened
Did you experience any change in your sexual performance after Rezum therapy?	0	1	2
Was your ability to ejaculate changed following Rezum therapy?	0	1	2

Table 5 contains the The International Prostate System Score.  

**Table 5:** International Prostate Symptom Score (I-PSS) From Barry et al [15].

In the past month:	Not at All	Less than 1 in 5 Times	Less than Half the Time	About Half the Time	More than Half the Time	Almost Always	Your score
1. Incomplete Emptying How often have you had the sensation of not emptying your bladder?	0	1	2	3	4	5	
2. Frequency How often have you had to urinate less than every two hours?	0	1	2	3	4	5	
3. Intermittency How often have you found you stopped and started again several times when you urinated?	0	1	2	3	4	5	
4. Urgency How often have you found it difficult to postpone urination?	0	1	2	3	4	5	
5. Weak Stream How often have you had a weak urinary stream?	0	1	2	3	4	5	
6. Straining How often have you had to strain to start urination?	0	1	2	3	4	5	
	None	1 Time	2 Times	3 Times	4 Times	5 Times	
7. Nocturia How many times did you typically get up at night to urinate?	0	1	2	3	4	5	
Total I-PSS Score							

Quality of Life Due to Urinary Symptoms	Delighted	Pleased	Mostly Satisfied	Mixed	Mostly Dissatisfied	Unhappy	Terrible
If you were to spend the rest of your life with your urinary condition just the way it is now, how would you feel about that?	0	1	2	3	4	5	6

	Delighted	Pleased	Mostly Satisfied	Mixed	Mostly Dissatisfied	Unhappy	Terrible
How satisfied were you with the results from Rezum therapy?	0	1	2	3	4	5	6

	Very Likely	Likely	Somewhat Likely	Mixed	Somewhat Unlikely	Unlikely	Very Unlikely
How likely would you be to recommend Rezum therapy to another patient with similar urinary symptoms?	0	1	2	3	4	5	6
